# Acetonemia and Vomiting in Children

**Published:** 1916-03

**Authors:** Howard Bucknell

**Affiliations:** Candler Building, Atlanta, Ga.


### ACETONEMIA AND VOMITING IN CHILDREN.

By Howard Bucknell M. D.

Vomiting is nature's excellent method of preventing an excessive amount of food from doing harm.

When irritants or excessive contents are ejected from the stomach vomiting should cease.

When vomiting does not cease after such causes are removed, or when the cause is unknown, the question of relief becomes urgent.

Among the more frequent causes of vomiting we find first, improper feeding; either food unsuitable for a particular child or spoiled food. Then, the onset of infectious diseases, such as scarlatina or pneumonia, also whooping cough, and the accumulation of mucus in the stomach from local causes or swallowed from the respiratory tract. Less frequently persistent vomiting is caused by brain lesions, meningitis, appendicitis, intestinal obstruction or pyloric stenosis. Other cases of vomiting are toxic in origin as, intestinal toxemia, uremia and acidosis· The last of these is by far the most important to physicians because of its great frequency either as a primary condition or as a complicating condition in other diseases.

During digestion ,carbohydrates are inverted from polysaccharids to mono-saccharids and as such are absorbed. The chief monosaccarid is dextrose· This is conducted by the portal circulation to the liver where the excess is stored as glycogen for future use. As the sugar in the blood and voluntary muscle is used, the stored glycogen in the liver is drawn upon to keep the sugar content of the blood constant. The absorption of Dextrose may be interfered with by starvation, vomiting, or loss of carbohydrate digestion.

High fever, as well as other anesthesia, rapidly removes. glycogen from the liver cells.

The body constantly needing fuel and an insufficient supply of glycogen being present, fat stored in the body is burned. Fat is normally oxidized to carbon dioxide and water.

When used too rapidly, part is converted into oxybutyric acid, which by oxidation forms aceto-acetic acid. This, by the loss of CO2, is converted into acetone· These substances are known as acetone bodies and when present in the blood cause the condition known as acetonemia, or acidosis, with serious symptoms, chief among which is vomiting.

When the vomiting stops voluntarily it is probably due to neutralization of the acetone bodies by alkaline substances in the body, elimination of acetone bodies and a diminished destruction of fat. The urine in untreated cases, is very acid and contains acetone bodies.

Acidosis may precede vomiting as in cylic vomiting or when the carbohydrate intake is insufficient. The food may, rarely, be too low in carbohydrates or, so high in some form of carbohydrate that the function of carbohydrate digestion may be for the time lost, and starvation result from an excessive supply. It may be a complication of infections or a result of lack of food.

In other cases the vomiting may result from another cause such as: food toxemia, swallowing bacteria laden mucus, improper feeding, certain food intolerances or pyloric stenosis; and an acidosis folow the resultant starvation as a complication·

Prolonged ether anesthesia, as has been shown by Dr. John Funke, robs the liver cells of glycogen. Vomiting is a frequent sequel as is the finding of acetone bodies in the urine.

In continued vomiting a urinary examination is of great value in determining why the vomiting persists· Of course, a careful physical examination should never be omitted. When the urinary examination is made, a test for acetone should be part of the routine. It would be astonishing to the physician who has neglected to make this test to see with what frequency it is positive, and it is also quite puzzling in some few cases, where we expect to find a positive reaction to find it absent. As a rule however, in all cases where vomiting persists for any considerable period acetone can be demonstrated. In cases of cyclic vomiting it is usual to find an acetone reaction, frequently accompanied by an indican reaction, present in increasing severity for a more or less extended period before the actual explosion occurs with its very severe type of vomiting. In the history of these cases there is usually found evidence of intestinal indigestion. Generally irregular feeding and a diet consisting largely of cereal carbo-hydrates, cane sugar and cream. The cereals and the cane sugar should be expected to furnish carbo-hydrates in such amount that acetonemia would not be present, but it is certainly the case that one kind of carbo-hydrate may not be well tolerated by certain types of children, while another is; and that while one sugar may cause trouble, another may be usefully supplied. It is to be noted that usually indican is present. It seems probable that these children have had carbo-hydrates in excess in a form not easily used by them· This excess has caused intestinal indigestion and intolerance for that particular form of carbo-hydrate, and has resulted in a relative carbo-hydrate insufficiency.

The statement is made in articles on this condition that there is little that can be done while the attack is in progress except to promote elimination, stop feeding and supply fluids, and that all efforts toward curing the condition should be made during intervals. This is certainly a mistake. Many children who persist in vomiting for days until the little patients are nearly pulseless and terribly prostrated, can quickly and easily have the vomiting and nausea relieved. They can get up fairly strong and active after a short, inconsequential illness by treating cases of acidosis for acidosis.

Kerley has made a great advance by using bi-carbonate of soda in these cases with his interval treatments.

During infections, especially when there is high fever, there is such a rapid use of body fuel that liver glycogen is insufficient to supply the demands, and body fats are used too rapidly, while the nausea and impaired digestive functions are barriers to the replenishment of the supply.

Where food toxemias are present we have an acute condition, not so very different in results, so far as the use of glycogen and fats is concerned, and inability to receive a fresh supply of carbo-hydrates, from the previous condition noted.

Bacteria laden mucus secretions are a potent factor in preventing the digestion and absorption of foods in proper amount, and therefore, cause also a carbo-hydrate starvation.

In chronic cases of malnutrition where one digestive function must over-work in order that even the minimum of life sustaining food may be used because the other digestive functions are temporarily greatly impaired, there is often a chronic carbohydrate insufficiency, which should be even graver than it is were it not for the fact that there is little fat to be destroyed, low temperature, and certainly a general decrease in metabolism. In pyloric stenosis the vomiting in itself with the inability of food to pass from the stomach into the intestinal canal creates a general starvation. Frequently when the condition is improved the vomiting continues even after food passes through the pyloris because of acetonemia, and by treating the acetonemia the patient may receive great benefit.

TREATMENT.

I.

When acetonemia is present with severe and persistent vomiting, body fluids are rapidly decreased by persistent loss of gastric juice, inability of the patient to take fluids, and the loss by respiration.

Water should be supplied by rectum if possible· If diarrhea is present, and it is impossible to have enemas retained, it should be supplied by hypodermoclysis, that the circulation may be kept up, the kidneys act freely, the intestinal contents kept from becoming too dry, and to dilute the toxic substances in the blood.

II.

Diacetic, oxybutyric, and beta oxybutyric acid are present in the blood. An alkali should be supplied to neutralize these substances as far as possible and facilitate their elimination.

III.

They are formed because the normal supply of carbo-hydrates is absent. This should be supplied in its most readily available form.

IV.

The vomiting reflex should be quieted. The procedure is as follows:

If the stomach contains irritating substances it is washed, the colon is washed, and a solution of sodium bicarbonate is left to be retained, if possible. A sedative is used to control the vomiting reflexes; usually by rectum. Bromides, chloral, or both combined may be used, however, chloretone has given me better results. If vomiting is not too severe, a solution of chloretone in appropriate amount may be given by mouth or through stomach tube after washing the stomach with bicarbonate of soda solution.

Dextrose solution 5 per cent in water is given next by rectum in most cases. This is alternated with bicarbonate of soda solution to be retained. Usually by the time the effect of the chloretone has disappeared the absorption of dextrose and bicarbonate of soda has decreased the nausea to such an extent that dextrose may be given by mouth. Usually vomiting stops altogether, and does not recur after a few hours of treatment. If it does recur the enemas are to be repeated. As soon as the vomiting stops, and a cathartic is given, if necessary to clean out the intestinal tract, feeding is resumed, unless otherwise contra indicated, and the attack is ended. One of the most frequent causes of severe vomiting in childhood is acetonemia. Children with acetonemia are likely to have persistent vomiting for a considerable period, or they may die, unless acetonemia is recognized and treated. Generally, the condition can be very promptly relieved, and, if it is not recognized, the results, especially in cases where another disease is present may be serious. In all cases with recurrent vomiting, or where vomiting is severe as a complication of other diseases, an acetone test should be made at the earliest possible moment, and if acetone is present, acetonemia should be treated as such no matter what other conditions are present. An acetone test should be a routine in all urinalyses for young children.

Cases of recurrent vomiting may be prevented in the vast majority of cases where this precaution is used. In many infections vomiting is a most serious complication. By keeping acidosis in mind many such complications are prevented, and when they occur may be made to assume a minor role. When using dextrose in treating children showing acetone reaction in the urine it should be used for effect and when this is obtained it should be discontinued. It is absolutely indicated as a pre anesthetic measure to supply the gylcogen which disappears from the liver cells when an ether anesthesia is given, and to prevent the weakening condition of the patient by post anaesthetic vomiting.

The following cases are used to illustrate treatment. A few only are used. No attempt is made to show all types of acidosis with vomiting either as a primary condition or complication:

M. L. D.—Girl eleven months of age. Family and personal history negative. Present condition very severe pyelitis. The urine on first examination showed so many pus cells that they formed a continuous deposit on slide from a shaken specimen. After treatment for this condition the progress was good and the condition began to clear up, but appetite was very poor and there was great difficulty in getting her to take sufficient nourishment. About three weeks after treatment was started she began vomiting and continued to do so at about forty minute intervals during the succeeding twenty-four hours. She was very much prostrated, was unable to retain even water. The urine had a distinct acetone reaction. She was given large doses of bicarbonate of soda by rectum, chloretone in water 1 1-2 gr. by mouth, giving a few drops at a time. Thirty minutes after finally administering the chloretone, she was given 1 drachm of powdered dextrose by mouth, a few grains at a time. This was followed by 8 ounces of 5 per cent solution of dextrose in plain water divided into two enemas of 4 ounces each, three hours apart. The dextrose was continued by mouth every 8 hours for the following two days. There was no more vomiting, the acetone cleared up, the child's strength was greatly improved and the treatment for pyelitis was continued with satisfactory results. The child was discharged cured.

In this case the pyelitis was very severe, but progress was being made toward recovery until it was interrupted by an acetonemia in which the resultant vomiting was so severe that it threatened to terminate fatally. Recognition and treatment of the complication was all that was necessary to correct the condition promptly.

C. W.—Boy two years of age. Whooping cough. Was doing fairly well when he had an acute dilatation of the stomach, with respiration of about 100, faint pulse that was almost uncountable and great prostration. The stomach was washed, the condition was relieved temporarily when he started persistent vomiting. He was unable to retain nourishment or water, and the urine showed a marked indican reaction with a very decided acetone reaction. He was given soda bicarbonate enema to be retained, 1 grain of chloretone by mouth, followed in thirty minutes by 1 drachm of dextrose by mouth, a few grains at a time, and when the nausea stopped, was given calomel and milk of magnesia, which he retained without vomiting. The dextrose was continued by mouth at six hour intervals for the following twenty-four hours, when he resumed his usual routine and had no more vomiting attacks, except an occasional one following a severe paroxysm of coughing.

J. W.—Girl twenty months old. Tonsilitis. Temperature 103 1-5, pulse 150, breathing rough, with a few coarse rales. Abdomen was considerably distended. There was a very offensive flatus. The breath had a strong acetone odor, tongue was coated. No urinary examination was made. The colon was first washed with asofetida enema. The stomach was washed with bicarbonate of soda solution. The throat and the temperature were treated symptomatically. Chlorctone was given to control the nausea. Bicarbonate of soda 1 drachm to 8 ounces of water was given by mouth in small amounts until all was taken. Two drachms of dextrose were given during the day. There was no more vomiting. Speedy recovery.

S. B.—Girl. At two years of age had a severe vomiting attack following a freight. This attack lasted for three days with great severity, and the child could not retain even water by mouth until the end of the second day, and then in very small amounts. She was greatly prostrated for some days after the attack. Soon afterward she appeared to be quite well, and had no symptoms indicating that she was ill, until suddenly, from no apparent cause, a second attack occurred about two months after the first. These attacks recurred at irregular intervals of from weeks to months. Generally for a day or two before the onset of an attack she would become gradually more languid, her eyes became circled, tongue coated, breath would have an acetone odor, and vomiting would start. It appeared in some cases that these attacks could be prevented by giving at the earliest appearance of symptoms a cathartic, followed by calomel. If the calomel preceded another laxative or was given alone the attacks were always immediately ushered in and with greater severity. This condition lasted for four years, during which time the child developed almost normally but was flabby and generally below par. At this time she was given the treatment advised by Kerley by Dr. Boynton, and her condition improved, but on limited diet she became weak and did not seem to be growing satisfactorily. When the condition was recognized as recurrent acidosis and was treated as such at the first symptoms of the beginning of an attack, and interval treatment continued, the attacks stopped, the first two within a few hours after they had started, and she has been free from all vomiting for the last three years in spite of the fact that she has gone through other illnesses, including infections.

E. J.—Girl four years of age. Had been having poor appetite for some months, with history of between meal feeding. Large amount of sugar and principally a carbohydrate diet. Two days before called to see the child she complained of chilly sensations and was drowsy, on the second day began to vomit, and contiued to do so at intervals of from twenty minutes to one hour without any relief. Examination showed that the child had follicular tonsilitis· The pulse was very weak, the abdomen was scaphoid, the head was slightly retracted, and the temperature was 103. She was given 6 oz bicarbonate of soda enema at once and 1 gr of chloretone by mouth. This was immediately vomited. In two hours after the first soda enema had been given the vomiting had continued without any relief whatsoever. Three grains of chloretone dissolved in two ounces of water were given by enema. In about thirty minutes the child seemed more comfortable and there was no more vomiting for the next three hours. At the end of this time six ounces of 5 per cent dextrose solution was given by enema. Shortly afterward she began to vomit again, though less severely and less frequently. The dextrose enema was repeated in four hours and small amounts of lemon albumen were given by mouth, without further vomiting. Two days later the child came to my office for routine interval treatment and appeared quite strong and active. Between the last dextrose enema given and the time of coming to the office she was given 3 drachms of dextrose and one drachm of soda bicarbonate in each twenty-four hours·

E. T.—Girl eight and one-half months of age. Had apparently thrived on a carbo-hydrate preparation until five months of age, when she started vomiting, had high fever and alternating attacks of constipation and diarrhea. At that time she weighed 14 1-2 pounds, according to the mother's statement. When brought to this office she weighed 11 pounds, nine ounces. She was emaciated, ribs were prominent, eyes were glassy, abdomen was flat, skin hanging in folds, and the mother stated that she vomited soon after each feeding and kept on until the next feeding period. That one ounce of milk preparation, that she was taking at the time, was as large a feeding as she could take without immediately vomiting the entire amount. Occasionally on these small feedings she would go for two or three feedings without vomiting, and then start again vomiting sour milk and mucus. This child was so ill that I expected she would die before they would get her back to the house. The stomach was washed with a soda bicarbonate solution, a five per cent dexirose enema was given and she was started on small feedings of partly skimmed citrated milk. The urine showed a very pronounced acetone reaction. This was a chronic case, and I believed at first was moribund. The urine was examined daily and the child treated symptomatically, and the acidosis controlled with dextrose and soda bicarbonate as it returned, and after a rather long drawn out treatment the child made a complete recovery.

L. L.—Girl age nine months. Had acute food toxemia, vomiting and purging every few minutes. I saw this case with Dr. C. E. Boynton, and the child was in complete collapse. He had her in a mustard pack, and had stimulated her and the purging had stopped. She had had a pint of normal salt solution by rectum, which had been retained. She was still vomiting. She was given one drachm of dextrose, a few grains at a time by mouth until one drachm was taken, alternating each small dose with a few drops of water. An 8 ounce soda bicarbonate enema was also given. In two hours this was repeated. Four hours later she was decidedly stronger, there was no vomiting, and she had a rapid recovery, though dextrose and soda bicarbonate had to be continued for the following two days.

S. E. Y.—Girl two years old. Still's disease. While being treated for this condition she became very constipated, the temperature went to 104 3-5, pulse was 162. Her throat was sore and there was nothing else that could be found on examination except scaphoid abdomen and a thready pulse. She was vomiting very frequently and was greatly prostrated. She was given chloretone solution as an enema to control the nausea. This was followed by soda bicarbonate enema, and powdered dextrose by mouth. As soon as the nausea ceased she was treated for the throat condition without any tendency to vomit.

L. O.—Boy three and one-half years of age· History of poor appetite, between meal feedings, excessive sugars and cream. Had recurrent vomiting attacks which usually lasted four or five days. He was seen on the second day of this attack. Could retain a small amount of water at a time and nothing else. He had large chronic tonsils, considerable glandular enlargement, and his abdomen was considerably distended. After washing the colon with bicarbonate of soda solution, he was given 2 grains chloretone in 2 ounces water as enema. The vomiting stopped promptly and he was given dextrose by mouth. Two days later he came to the office. The urine on examination was very acid with extremely marked reaction for indican and acetone· By putting the child on proper diet and interval treatment there has been no recurrence in the past five months.

Candler Building, Atlanta, Ga.

